# Advancing Single-Molecule Biophysics: Next-Generation Organic Fluorophores with Tailored Labeling Strategies

**DOI:** 10.1021/cbmi.5c00007

**Published:** 2025-04-01

**Authors:** Lei Zhang, Dongwen Shen, Jiazhen Yang

**Affiliations:** School of Life Science and Technology, Key Laboratory of Developmental Genes and Human Disease, 12579 Southeast University, Nanjing, 210000, China

**Keywords:** organic fluorophores, photostable, photoswitchable, photoactivatable, super-resolution microscopy, single-molecule fluorescence microscopy, single-molecule FRET, biolabeling

## Abstract

Recent advancements in single-molecule biophysics have been driven by breakthroughs in advanced fluorescence microscopy techniques and the development of next-generation organic fluorophores. These cutting-edge fluorophores, coupled through tailored biolabeling strategies, offer single-molecule brightness, photostability, and phototunability (i.e., photoswitchable, photoactivatable), contributing to enhancing spatial and temporal imaging resolution for studying biomolecular interactions and dynamics at single-event precision. This review examines the progress made over the past decade in the development of next-generation fluorophores, along with their site-specific labeling methods for proteins, nucleic acids, and biomolecular complexes. It also explores their applications in single-molecule fluorescence-based dynamic structural biology and super-resolution microscopy imaging. Furthermore, it examines ongoing efforts to address challenges associated with fluorophore photostability, photobleaching, and the integration of advanced photophysical and photochemical functionalities. The integration of state-of-the-art fluorophores with advanced labeling strategies aim to deliver complementary correlative data, holding promise for revolutionizing single-molecule biophysics by pushing the boundaries of temporal and spatial imaging resolution to unprecedented limits.

## Graphical abstract

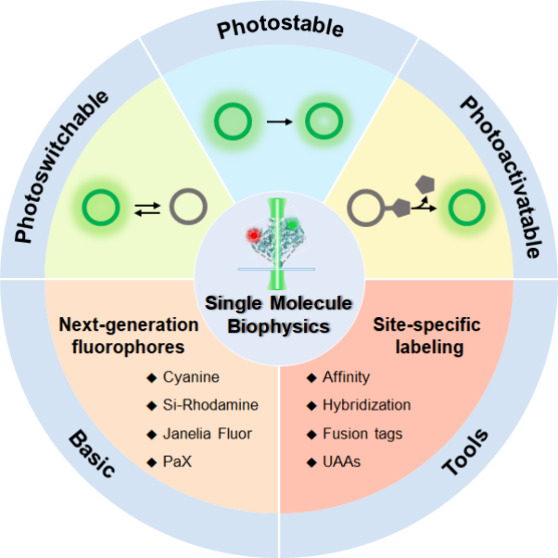

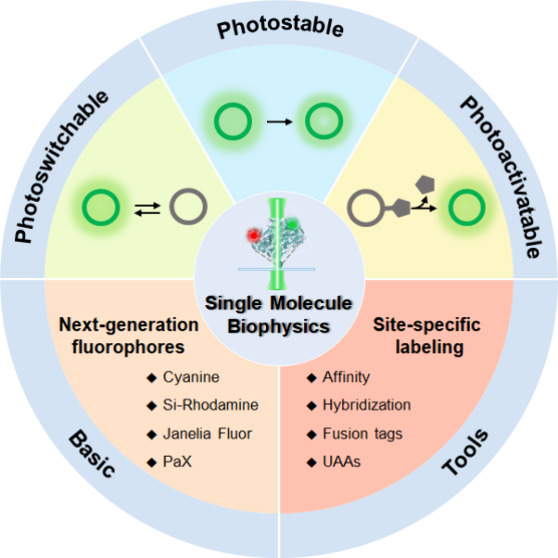

## 1 Introduction

Static electron microscopy provides atomic-resolution snapshots of molecular processes but is often insufficient on its own for capturing the time-dependent evolution of dynamic interactions and conformational changes.^1^ Single-molecule biophysics has revolutionized our understanding of these dynamic processes by enabling real-time observation and precise quantification of molecular interactions and structural changes.^2^ Single-molecule techniques first emerged as a powerful biophysical approach in the recordings of single-ion channel activity using patch-clamp in the 1970s.^3^ Since then, the field of single-molecule biophysics has significantly expanded, encompassing studies on protein folding and dynamics,^4–6^ ribosome molecular machine,^7,8^ pre-mRNA splicing,^9^ conformational transitions of membrane transporters and receptors,^10–14^ and CRISPR-associated endonucleases along with protein-nucleic acid interactions.^15^ This progress has been enabled by transformative physical techniques,^16^ including atomic force microscopy,^17^ optical tweezers,^18^ single-molecule FRET microscopy,^19–21^ and super-resolution fluorescence microscopy.^22^ Among these, fluorescence microscopy-based methods stand out for their accessibility, versatility, and widespread adoption across scientific disciplines. Unlike ensemble fluorescent measurements, which average signals across large populations of molecules, single-molecule fluorescence approaches require highly reliable signals distinctly resolved from background noise to accurately capture the unique behaviors of individual molecules.^23^

Central to these methods are reporter fluorophores, which must exhibit precise localization to the target molecule with minimal functional disruption, high brightness, exceptional photostability, and chemical and photophysical tunability to meet the specific demands of single-molecule fluorescence microscopy.^24^ In some cases, two or more fluorophores with distinct emission spectra are employed, enabling simultaneous monitoring of multiple events and multiplex imaging.^25^

However, inherent photobleaching, unpredictable blinking, and the persistent “always-on” state of fluorophores pose significant challenges,^26^ as many single-molecule super-resolution techniques rely on fluorophore switching for precise localization,^27^ limiting the temporal and spatial resolution of single-molecule imaging. These limitations hinder the ability to capture a full spectrum of molecular behaviors, from rapid conformational changes to complex multistep processes. As a result, there is a growing demand for next-generation fluorophores specifically designed to overcome these obstacles and enable reliable single-molecule studies without compromising critical data.

Next-generation fluorophores are highly sought after for their enhanced brightness, photostability, and advanced single-molecule photoswitching and photoactivation capabilities.^28^ The brightness of a fluorophore, governed by its extinction coefficient and quantum yield, largely depends on the optimization of its conjugated core structure and electron-withdrawing/donating groups. Enhanced photostability enables resistance to photobleaching, ensuring consistent signal strength over extended periods and allowing continuous observation of individual molecular trajectories. Single-molecule photoswitching or photoactivation capabilities add further versatility by allowing precise control over fluorescent on and off states, a feature particularly valuable in super-resolution microscopy where accurate localization of individual fluorophores is critical. Several review articles have summarized fluorophore advances in this field.^29–32^ For instance, Zheng et al. (2014) provided an overview of the fundamental photophysical requirements for organic fluorophores employed in single-molecule fluorescence imaging,^31^ whereas Vaughan^29^ and Zhang^30^ et al. (2018 and 2023) systematically discussed the strategies and applications of photoactivatable fluorophores in super-resolution microscopy.

How these fluorophores can be noninvasively connected to the biological target of interest—a process known as biolabeling—is another crucial consideration. The optimal labeling strategy depends on the nature of the target molecule, whether it is a protein, a nucleic acid, or a protein-nucleic acid complex. For proteins, labeling often relies on fluorophore-labeled antibodies or nanobodies through immunofluorescence approaches.^33^ To capture more detailed structural dynamics or interactions, site-specific amino acid mutations,^34^ genetically encoded unnatural amino acids (UAAs),^35^ or engineered protein and peptide tags are commonly employed.^36^ These tags facilitate labeling through noncovalent affinity interactions or covalent chemical bonds, frequently leveraging via copper-catalyzed or copper-free click chemistry reactions.^37,38^ For nucleic acids (DNA or RNA), traditional biotin–streptavidin labeling involves fluorescently labeled streptavidin binding to biotinylated target nucleic acids.^39^ Chemical modifications can introduce reactive groups, such as amines, thiols, or alkynes, into nucleotides, allowing conjugation with fluorescent dyes through complementary reactive sites.^40^ Aptamer-based labeling utilizes engineered RNA aptamers like Spinach, Broccoli, Mango, and Okra, which bind to specific fluorophores and enhance their fluorescence upon interaction.^41^ Single-molecule fluorescence in situ hybridization (smFISH) achieves superior specificity by using fluorescently labeled oligonucleotide probes targeting multiple regions of nucleic acids, enabling visualization of individual RNA or DNA molecules at single-molecule resolution within fixed cells.^42^ For protein-nucleic acid complexes, the labeling strategy depends on the domain of interest, employing the corresponding methods described above for proteins or nucleic acids. These labeling approaches establish a critical connection between the biotarget and the fluorophore reporter, facilitating the in situ tracking of molecular interactions, kinetics, and structural dynamics.

The objective of this review is to examine recent advancements in the development of next-generation organic fluorophores, their site-specific labeling techniques, and their optimization for single-molecule biophysics applications to enhance temporal and spatial resolution (Scheme 1). This review provides a comprehensive analysis of key technical developments in next-generation fluorophores, focusing on their enhanced brightness, photostability, photoswitching, and photoactivation capabilities tailored for single-molecule and super-resolution microscopy-based applications. Furthermore, it examines tailored site-specific labeling strategies, including noncovalent affinity coordination, covalent conjugation, fusion tags, and transient hybridization, and evaluates how these advancements in fluorophore properties and labeling precision have significantly improved imaging resolution in single-molecule studies. By exploring these developments, the review underscores how these innovations enable researchers to observe and analyze dynamic molecular processes with unprecedented detail, thereby driving progress in the field of single-molecule biophysics.

**Scheme 1:**
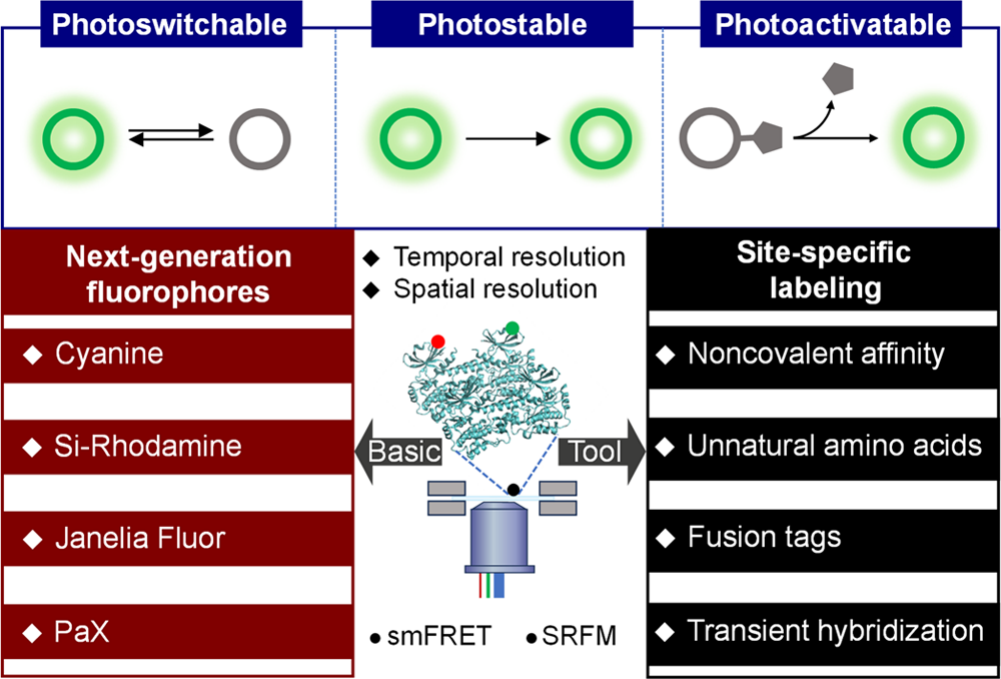
An Overview Schematic Framework Illustrating Recent Advancements in Next-Generation Fluorophores, Emphasizing Their Photophysical and Photochemical Properties at the Single-Molecule Level and Strategies for Site-Specific Biolabeling^a^

a These developments aim to improve temporal and spatial resolution in single-molecule biophysical studies, including applications in single-molecule Förster resonance energy transfer (smFRET) and super-resolution fluorescence microscopy (SRFM).

## 2 SINGLE-MOLECULE FLUORESCENCE TECHNIQUES IN BIOPHYSICS

Fluorescence techniques, including fluorescence spectroscopy and microscopy, have seen significant advancements in biophysics since the 1990s.^43^ These developments, coupled with innovations in excitation lasers, detectors, cameras, surface tethering methods, and data collection techniques, have facilitated the transition from ensemble to single-molecule measurements. Techniques such as single-molecule fluorescence resonance energy transfer (smFRET)^44^ and total internal reflection fluorescence (TIRF) microscopy^45^ have become indispensable for detecting single-molecule interactions and conformational changes with high spatial and temporal resolution. The emergence of super-resolution fluorescence microscopy, sometimes referred to as “nanoscopy”^46^ for its ability to achieve nanometer-scale resolution, has significantly enhanced observational capabilities by surpassing the diffraction limit of conventional optical microscopy.^47^ These advanced methodologies can be broadly classified into three principal categories—structured illumination microscopy (SIM),^48^ stimulated emission depletion (STED) microscopy,^49^ and single-molecule localization microscopy (e.g., stochastic optical reconstruction microscopy (STORM),^50^ photoactivated localization microscopy (PALM),^51^ and DNA point accumulation for imaging in nanoscale topography (DNA-PAINT)^52^)—along with the integration of MINFLUX,^53^ which combines the advantages of STED and single-molecule localization, further optimizes photon usage and dramatically increases spatial and temporal resolution. Next-generation organic fluorophores and corresponding site-specific biolabeling strategies have been developed to enhance signal stability and controllability, enabling more detailed investigations of single-molecule dynamics, molecular interactions, and structural transitions.

Despite their advantages, advanced fluorescence imaging techniques face several challenges. Photobleaching remains a significant limitation, as prolonged illumination sharply decreases the number of photons emitted by a single fluorophore, reducing signal strength and hindering long-term observations. Irregular blinking of fluorophores—caused by unpredictable transitions to the triplet state (T_1_)—complicates data interpretation by introducing interruptions in signal continuity. Furthermore, single-molecule localization-based super-resolution microscopy techniques rely on photoswitchable or photoactivatable fluorophores capable of frequent “on” and “off” transitions to achieve precise localization of individual molecules. High biolabeling specificity with these fluorophores is critical to ensure that fluorescence signals accurately represent the behavior of the target molecule. Addressing these challenges requires advancements in fluorophore design, labeling strategies, and customization for specific targets to improve the reliability and resolution of single-molecule studies. Moreover, with single-molecule instrument development approaching technological saturation, the expanding frontiers of bioimaging impose increasing demands on the range of available fluorophores.

## 3 NEXT-GENERATION ORGANIC FLUROPHORES FOR SINGLE-MOLECULE BIOPHYSICS

Ideal fluorophores for single-molecule studies should stably emit a sufficient number of photons over a series of frames to ensure accurate detection and precise localization (Figure 1). In principle, upon illumination, a fluorophore is expected to rapidly cycle between the excited singlet state (S_1_) and the ground state (S_0_), resulting in consistent photon emission. However, deactivation from S_1_ into nonfluorescent (“dark”) states is frequently observed, resulting in fluorophore instabilities driven by photophysical and photochemical processes associated with these states.^54^ In addition to radical or cis–trans isomerized forms (depending on the fluorophore structure), a common deviation is the nonfluorescent triplet excited state (T_1_), which a fluorophore enters from S_1_ via intersystem crossing.^55^ Although the quantum yield of the triplet state in organic fluorophores used for single-molecule imaging is typically low (often less than 0.01),^56,57^ its higher energy relative to the ground state and extended lifetime (10^–6^ to 10^–4^ seconds)^57,58^ significantly impact fluorophore performance. In contrast, the S_1_ state decays to the S_0_ state through radiative fluorescence relaxation pathways on time scales of 10^–10^ to 10^–9^ seconds.^59^ Transitions to a long-lifetime T_1_ state can result in either photobleaching, causing irreversible damage and necessitating strategies to mitigate T_1_ for enhanced photostability and signal consistency in single-molecule biophysical applications, or reversible recovery to S_0_ mediated by buffer additives. facilitates reversible photochemical switching, essential for single-molecule localization microscopy (SMLM), albeit with reduced brightness and signal-to-noise ratio.^60,61^ In SMLM, most fluorophores undergo photoswitching to a long-lived OFF state, such as a triplet or a photochemically induced nonfluorescent dark state such as photoisomerization. Stochastic reactivation of a small subset into the emissive S_1_ state ensures spatial separation beyond the diffraction limit, allowing precise localization by fitting their photon distribution to a point spread function.

**Figure 1:**
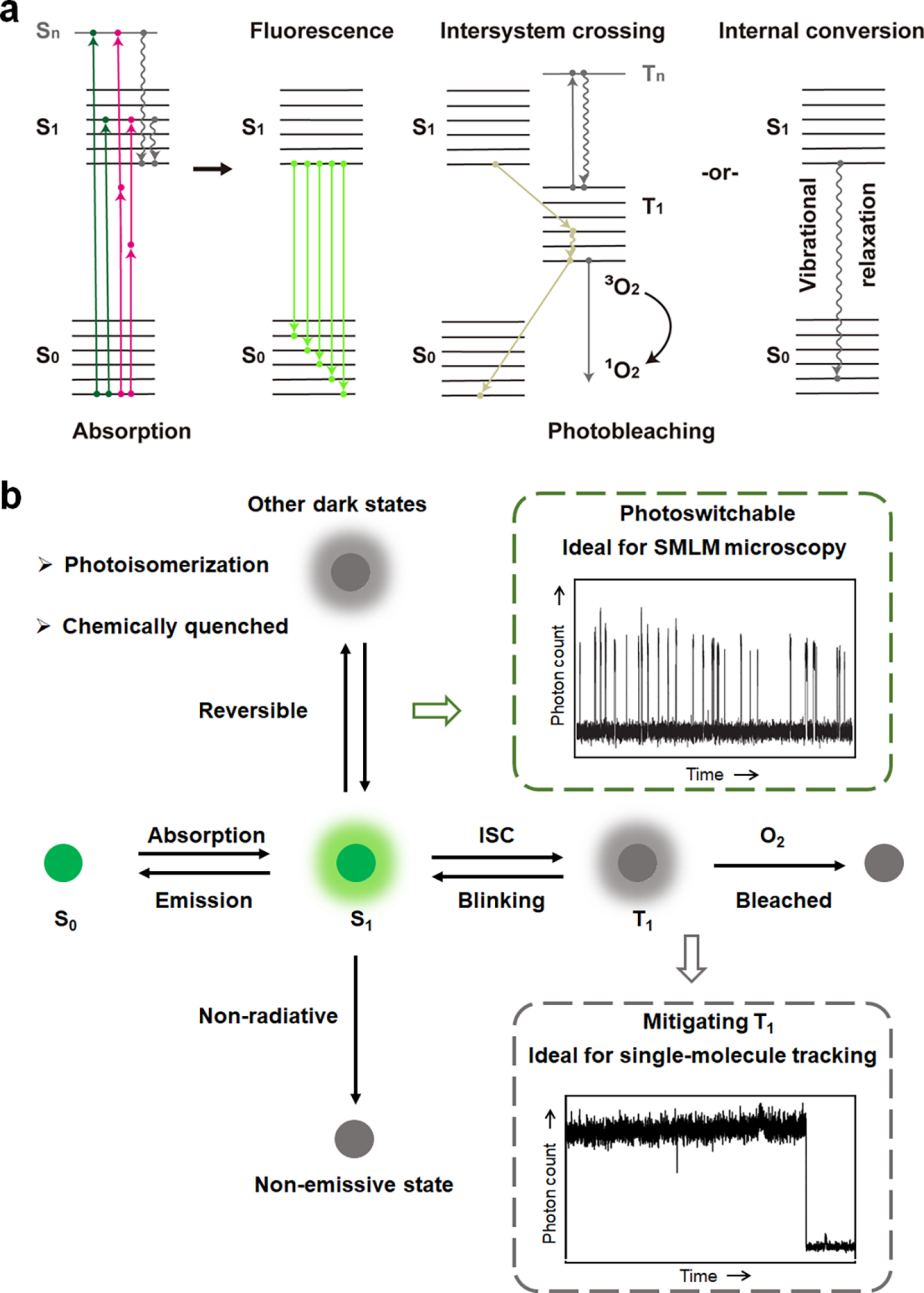
(a) Schematic representation of the energy state diagram illustrating fluorophore excitation and deactivation pathways. The diagram highlights radiative transitions ( fluorescence) and nonradiative transitions (intersystemcrossing (ISC) and internal conversion) between energy states. S_0_: the singlet ground state; S_1_: the first excited singlet state; S_2_: the second excited singlet state; T_1_ and T_n_: the first and n^th^ excited triplet states. Dashed arrows denote nonemissive relaxation pathways, which do not emit photons but critically influence the photophysical behavior of fluorophores. (b) Representative idealized time traces of fluorophore emission signals observed in single-molecule fluorescence studies. The traces showcase fluorescence photoswitching and photobleaching events, which are characteristic behaviors of a fluorophore in single-molecule studies. Photoisomerization: light-induced isomerization observed in certain fluorophores observed in certain fluorophores (e.g., cyanine family), typically initiated from an excited or intermediate electronic state.

Consequently, next-generation organic fluorophores (Table 1) are designed to exhibit sufficient brightness for robust detectability, exceptional photostability for single-molecule tracking, and optimized photoswitchability and photoactivability for compatibility with single-molecule fluorescence and localization-based super-resolution microscopy.

**Table 1:** Next-Generation Fluorophores for Single-Molecule Biophysics

**Fluorophore**	**Labeling Method**	**Key Characteristics**	**Applications**
**Cy5B**	Maleimide/NHS-ester/Click chemistry	Far-red, photostable	Light-sheet dSTORM^62,63^
**Alexa Fluor 647**	Maleimide/NHS-ester/Click chemistry	High brightness, photoswitchable	General STORM^64–66^
**Atto 647N**	Maleimide/NHS-ester/Click chemistry	High brightness, photostable	STED microscopy^67^
**CF680**	Maleimide/NHS-ester chemistry	Far-red, minimal photobleaching	STORM, deep-tissue imaging^68^
**JF**_**549**_	HaloTag/SNAP-tag ligand	Bright, photostable, cell-permeable	Live-cell single-molecule tracking^69–73^
**JF**_**646**_	HaloTag/SNAP-tag ligand	High brightness, photostability, spontaneous blinking	Single-molecule tracking, STED microscopy^72–74^
**Photoactivatable JF dyes**	HaloTag/SNAP-tag ligand	UV–visible photoactivation	PALM, single-molecule tracking^73^
**JF**_**635**_ **b**	HaloTag/SNAP-tag ligand	Intermediate duty cycle, spontaneous blinking	Live-cell STORM^75^
**Yale676sb**	SNAP-tag, NHS-ester chemistry	High brightness, photostability	Single-molecule tracking^76^
**HMSiR dyes**	HaloTag/SNAP-tag ligand	Spontaneously blinking	Live-cell dSTORM imaging^77^
**PK Mito**	NHS-ester/Maleimide chemistry	Minimal phototoxicity, mitochondrial targeting	Long-term mitochondrial dynamics^78–80^
**SF8(D4)**_**2**_	NHS-ester chemistry	Rotaxane protection, photostability	High-resolution single-molecule imaging^81^
**HBC**	Noncovalent RNA aptamer interaction	>3000-fold fluorescence increase	RNA localization^82^
**NBSI**	Noncovalent RNA aptamer interaction	Large Stokes shift, bright fluorescence	RNA localization^83^
**PaX dyes**	NHS-ester chemistry, HaloTag ligand	Ultraphotostable, high photon output	MINFLUX nanoscopy^84,85^
**Cy3B, Atto 655**	Transient DNA hybridization	Controllable blinking, high photon budget	DNA-PAINT super-resolution imaging^86–89^
**JFX669**	HaloTag ligand	Photoconvertible fluorophore, visible-light activation	PALM super-resolution imaging^90^
LD555 & LD655	Engineered cysteine	Brightness, minimal photobleaching	smFRET for protein dynamics^91,92^
Cy5B & Dy-751	Engineered cysteine	Photostable FRET pair, excellent photon budget	smFRET for conformational kinetics^93^
Alexa Fluor 546 & Alexa Fluor 647	NHS-ester chemistry	Commercially available smFRET pair	smFRET for protein dynamics^94^
Alexa Fluor 488 & Atto 643	NHS-ester chemistry	Minimal spectral overlap	smFRET for GPCR dynamics^95^
Cy3B & LD650 & LD750	Engineered cysteine	High brightness, photostable	Multicolor smFRET^96^

### 3.1 Sufficient Brightness

Fluorophores with a high extinction coefficient (>50,000 M^–1^ cm^–1^) and a moderate quantum yield (>0.1) are considered ideal for single-molecule fluorescence studies.^97^ However, traditional cyanine dyes, such as trimethine cyanine (Cy3, Φ_F_ = 0.09) and pentamethine cyanine (Cy5, Φ_F_ = 0.15) in aqueous environments, exhibit limited quantum yields due to nonemissive deactivation pathways involving polymethine isomerization.^59^ Enhancements to cyanine dyes have focused on overcoming their intrinsic photophysical limitations through structural modifications. Strategies include extending conjugated π-systems,^86^ replacing regular water (H_2_O) with heavy water (D_2_O) —particularly for Alexa Fluor 647—due to reduced nonradiative transition pathways,^98–100^ introducing sulfonate groups to improve solubility and prevent dye aggregation,^101,102^ coupling dyes with plasmonic metallic structures,^103^ and leveraging supramolecular interactions with cucurbituril host molecules.^104^ Notably, stabilizing the π-conjugated system of Cy3 by incorporating six-membered rings into the polymethine chain led to the development of Cy3B, which exhibited a substantially higher quantum yield (Φ_F_ = 0.85) compared to traditional Cy3.^86^ Building on this success, the Schnermann lab extended the strategy to pentamethine and heptamethine derivatives, producing advanced dyes such as Cy5B (Figure 2a, Φ_F_ = 0.69, compared to Φ_F_ = 0.15 for Cy5), which also demonstrated extended fluorescence lifetimes.^62,63^ which demonstrated superior labeling efficiency and enhanced compatibility with single-molecule FRET studies and 3D lattice light-sheet dSTORM localization microscopy.^101,102^

**Figure 2:**
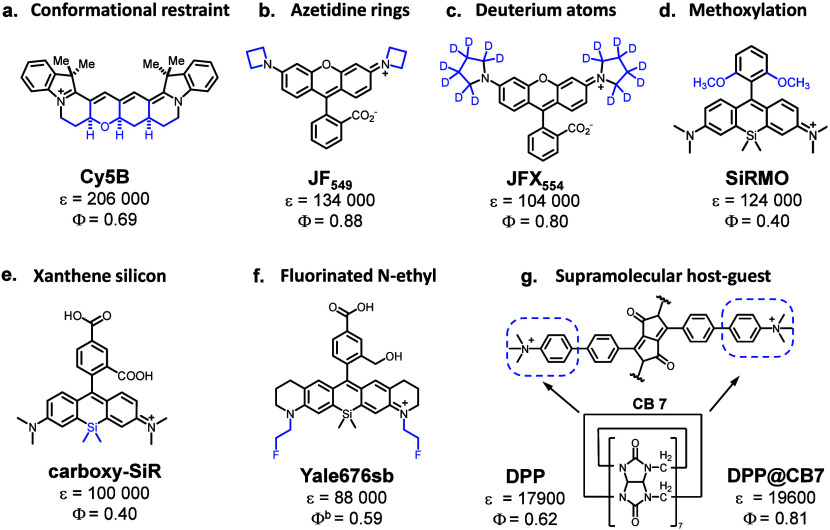
Representative strategies and molecular structures designed to improve fluorescence brightness for single-molecule fluorescence imaging. (a) Conformational restraint - Structural modifications that rigidify molecular frameworks, minimizing nonradiative decay pathways and enhancing quantum yield. (b) Azetidine rings - Incorporation of azetidine groups to reduce torsional freedom, suppress vibrational relaxation, and increase brightness. (c) Deuterium incorporation - Substitution of hydrogen with deuterium to reduce vibrational quenching, extending fluorescence lifetime and enhancing brightness. (d) Methoxybenzene modifications - Addition of methoxy groups to aromatic rings, stabilizing excited states and improving quantum efficiency through electron-donating effects. (e) Xanthene silicon substitution - Replacement of oxygen atoms with silicon in xanthene dyes, resulting in red-shifted spectra and enhanced brightness via extended π-conjugation. (f) Fluorinated N-ethyl groups - Introduction of fluorinated alkyl substituents to optimize electron density, suppress nonradiative losses, and boost photostability. (g) Supramolecular host–guest chemistry - Encapsulation of fluorophores via host–guest interactions, shielding them from quenchers and improving fluorescence brightness and stability. Blue regions in the chemical structures highlight specific sites of modification intended to optimize the brightness of the conjugated fluorophore.

For xanthene dyes, particularly rhodamines, several strategies have been developed to enhance brightness for single-molecule fluorescence biophysical applications (Figure 2b–g).^105^ Key approaches include substituting the *N*,*N*-alkyl groups with nitrogen-containing azetidine rings (Figure 2b),^106^ deuterium atoms (Figure 2c),^107^ or fluorinated pendant phenyl ring (Figure 2f).^76^ These modifications mitigate nonradiative decay pathways by suppressing twisted intramolecular charge transfer (TICT) and restricting C–N bond rotation, thereby enhancing the fluorescence pathway. Further improvements have been achieved by replacing the xanthene oxygen atom with heteroatoms such as silicon (Figure 2d–f) and introducing azetidine groups, which collectively reduce nonradiative decay and enhance photophysical properties.^69–71,108^ Additionally, modulating the lactone-zwitterion equilibrium and stabilizing the zwitterionic form through methylation or methoxylation (Figure 2d) significantly enhances quantum yield.^109^ Another indirect approach to improving visual brightness involves optimizing red-shifted rhodamine dyes to minimize cellular autofluorescence at shorter wavelengths.^110,111^ These strategies above have culminated in the development of optimized fluorophores, including Yale676sb by the Schepartz lab,^76^ carboxy-SiR by the Johnsson lab,^108^ and JF_549_ and JFX_554_ by the Lavis lab.^69–71^ All these modifications have contributed to optimizing the brightness of the rhodamine family and enhancing their suitability for single-molecule-based imaging applications.

The development of fluorophores with high brightness continues to enhance the toolkit for single-molecule imaging.^112–115^ Oxazine-based Atto dyes (www.atto-tec.com), with rigid structures and high quantum yields (Φ_F_ = 0.60–0.90), achieve exceptional brightness, particularly Atto 647N and Atto 655.^67^ Trianguleniums,^116^ another class of fluorophores, are planar and rigid carbocations with molar absorption coefficients of 15,000–20,000 M^–1^·cm^–1^ and fluorescence lifetimes up to 23 ns—far exceeding the <5 ns lifetimes typical of mainstream dyes.^117^ In 2021, Kacenauskaite et al. introduced a dyad combining a perylene antenna for high absorption and a triangulenium emitter. This design enhanced brightness by up to 5-fold, extended fluorescence lifetimes (∼17 ns), and achieved high quantum yields (Φ_F_ = 0.75).^118^ More recently, Kim et al. (2023) demonstrated fluorescence enhancement of DPP (diketopyrrolopyrrole) fluorophore via supramolecular host–guest complexation with cucurbit[7]­uril (CB7) (Figure 2g).^119^ The CB7 complex effectively stabilized DPP by reducing nonradiative decay and aggregation-induced quenching, while simultaneously improving water solubility and molecular rigidity. These efforts have progressively enhanced the brightness of fluorophores for single-molecule fluorescence imaging studies.

### 3.2 Single-Molecule Fluorophore Photostability

Photoinstabilities, including blinking and photobleaching, primarily originate from the triplet excited state of fluorophores, often followed by oxidation processes.^31^ Specifically, singlet oxygen and other reactive oxygen species generated through dye sensitization can oxidize fluorophores via multiple mechanisms. Additionally, the triplet excited state itself can undergo various photooxidation reactions. To enhance fluorophore photostability and thereby increase the photon budget for single-molecule imaging experiments, three prominent strategies have been developed. First, structural modifications, such as incorporating electron-donating or withdrawing groups and heavy atom substitutions, have beenemployed to reduce the fluorophore’s susceptibility to reactive oxygen species, minimize side reactions, and slow photobleaching. Second, optimization of imaging conditions, including the use of oxidant buffer systems or oxygen-scavenged environments, has been shown to significantly reduce photooxidative damage. For example, the study by the Luin lab^120^ advanced two-color single-molecule tracking by systematically optimizing fluorophore combinations and imaging conditions. The authors optimized the Atto 488 & Atto 565 fluorophore pair, a low-autofluorescence N-PK51 cover glass, and an optimal combination of Trolox (a triplet-state quencher) and *n*-propyl gallate (a reactive oxygen species scavenger), which significantly enhanced the signal-to-noise ratio in multicolor single-molecule imaging. Third, reducing the triplet-state lifetime, either through the introduction of triplet-quenching molecules into the imaging buffer or by conjugating intramolecular triplet quenchers (Figure 3a–f,i), effectively suppresses the formation and reactivity of the triplet state.^54,120,121^ Other strategies for enhancing photostability, including engineered protective molecular shielding and dye-doped nanoscale systems, were summarized in the 2020 review by Demchenko et al.^122^

**Figure 3:**
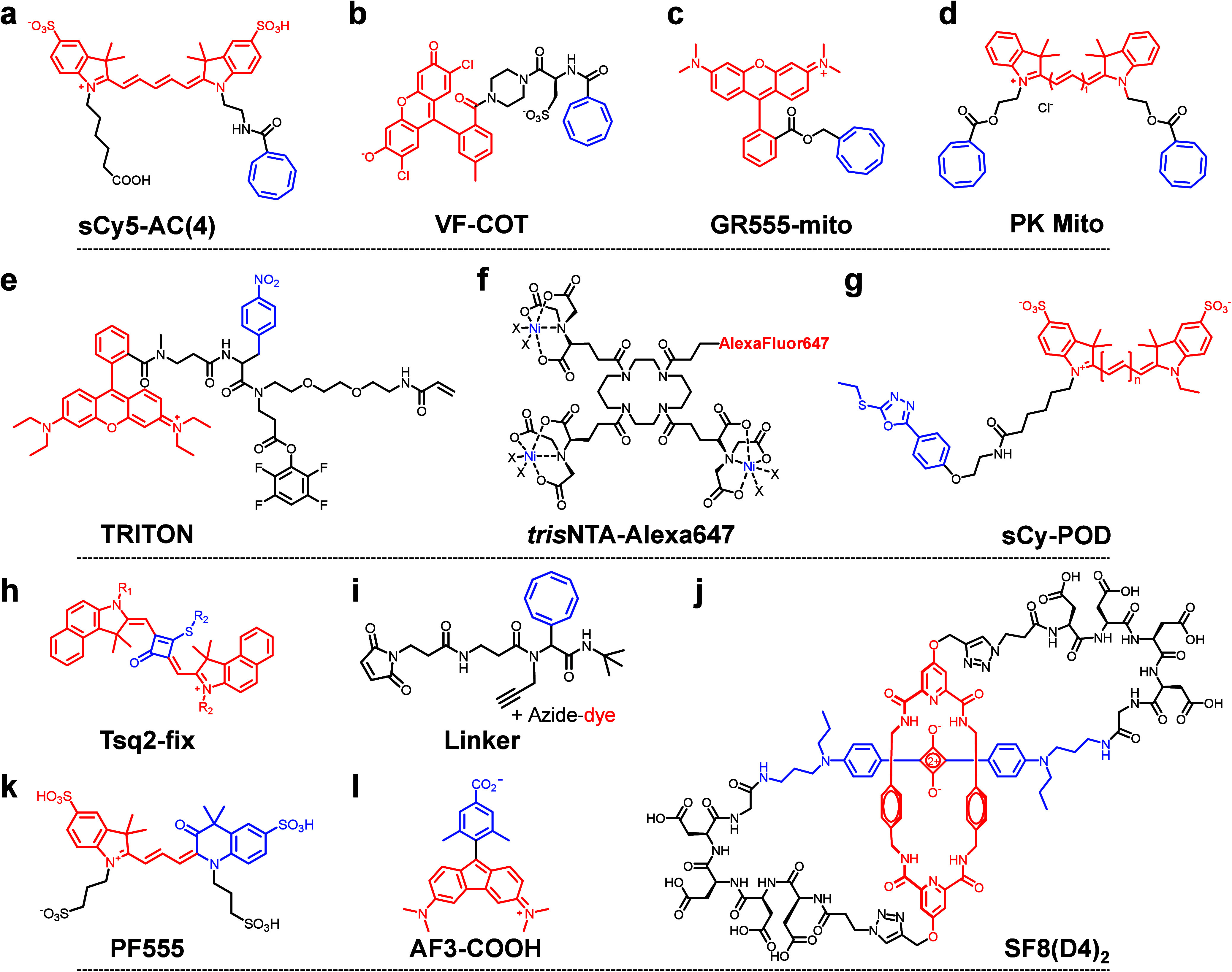
Representative fluorophore structures designed to improve photophysical stability for single-molecule fluorescence imaging. (a–f) Self-healing fluorophores with conjugated triplet-state quenchers, including COT (a, b, c, d), NPA (e), and *tris*NTA (f). (g) Cyanine fluorophores conjugated with electron-deficient aromatic thioether POD. (h) Squaraine dyes (Tsq2-fix) incorporating a thiocarbonyl group. (i) Self-healing linker with conjugated triplet-state quencher of COT, fluorophore tag and bioconjugation site. (j) Macrocyclic structure of SF8­(D4)_2_ dye. (k) PF555 fluorophore characterized by a unique 3-oxo-quinoline-substituted asymmetric cyanine structure. (l) Aminofluorene (AF) dyes leveraging ground-state antiaromaticity to improve photophysical properties. The red and blue regions denote the core fluorophores and chemical modifications intended to enhance photostability.

Focusing on structural modifications, several research groups have significantly advanced fluorophore design to enhance photostability for single-molecule florescence imaging. The Johnsson lab developed bright, photostable silicon-containing rhodamine derivatives equipped with diverse labeling-reactive groups.^123^ The Lavis lab introduced the azetidine strategy, resulting in Janelia Fluor dyes with enhanced brightness and photostability, widely applicable to live-cell single-molecule imaging.^106^ The Urano lab developed the SaraFluor B series fluorophores (corresponds to HMSiR), which exhibit significantly improved photostability compared to traditional tetramethylrhodamine (TMR) fluorophores. More recently, Kniazev et al. (2023) presented the photostable SF8­(D4)_2_ fluorophore, designed using macrocyclic rotaxanes to shield the squaraine core from oxidative damage and nucleophilic attack (Figure 3j).^81^ In 2024, Liu et al. developed a thiolation strategy for squaraine dyes (Tsq2-fix), substituting the central cyclobutene with a thiocarbonyl group (Figure 3h).^124^ This modification increased the photobleaching lifetime 5-fold compared to traditional oxo-squaraines and doubled that of Cy5. Xu and Liu’s groups advanced high-brightness, high-sensitivity fluorophores by regulating twisted intramolecular charge transfer (TICT).^125^ The Zhang group (2024) introduced aminofluorene (AF) dyes (Figure 3l), a novel class of small-molecule fluorophores that utilize ground-state antiaromaticity to achieve enhanced photostability under high-power irradiation and tunable emission spectra spanning 700–1600 nm.^126^ In 2025, the Ryu group developed Phoenix Fluor 555 (PF555, Figure 3k), a superphotostable organic dye featuring a distinctive 3-oxo-quinoline-substituted asymmetric cyanine structure. PF555 offers an order-of-magnitude longer photobleaching lifetime compared to conventional dyes, enabling extended live-cell single-molecule imaging of dynamic processes, such as EGFR endocytosis, under physiological conditions without the need for antiphotobleaching additives.^127^

Another focus in photostable fluorophore development is the triplet-state quenching strategy,^121,128–130^ particularly through the incorporation of the photostabilizer cyclooctatetraene (COT) derivatives^131–133^ (Figure 3a–d), resulting in “self-healing fluorophores”.^134–137^ The Blanchard group demonstrated that introducing COT into Cy5 fluorophores reduced the triplet-state lifetime from 63 to 1.1 μs, significantly increasing the number of photons emitted before photobleaching.^138^ A comprehensive review of this concept was provided by the Cordes group in 2021.^139^ Cordes and collaborators, including our group, developed self-healing linkers^140^ (Figure 3i) and multifunctional fluorophores^141^ using Ugi-4CR scaffold synthesis, offering a versatile strategy to enhance the functionality of commercial fluorophores—such as improved photostability for photobleaching-resistant single-molecule FRET and long-term super-resolution imaging.^140^ The Hofkens lab (2022) introduced stabilizer-conjugated TRITON fluorophores to enhance photostability in expansion fluorescence microscopy.^142^ In 2023, the Chen lab developed photostabilizer-functionalized gentle rhodamines optimized for cellular and subcellular imaging.^78,79^ They identified rhodamine GR555 and cyanine PK Mito, which exhibit reduced phototoxicity, minimized singlet oxygen generation, and robust photostability via strategic conjugation of COT. They also introduced thiol bioconjugation strategies incorporating electron-deficient aromatic thioether phenyloxadiazole (POD) linkage (Figure 3g), achieving a 1.5- to 3-fold increase in total photon counts of cyanine dyes.^80^ In 2024, the Liu group elucidated the multifaceted photophysical effects of COT on self-healing fluorophores, highlighting its dual role in quenching triplet states and influencing singlet-state dynamics via energy transfer to dark states and photoinduced electron transfer.^132^ They proposed the ΔE descriptor as a predictive tool for optimizing photostability and mitigating adverse effects. Beyond the use of COT as a triplet-state quencher, Glembockyte and Cosa et al. demonstrated the effectiveness of Ni^2+^ (2015)^143^ and thio-imidazole amino acids (2023)^144^ as triplet state quenchers to improve the photostability of cyanine fluorophores, while in 2018, they introduced *tris*NTA-modified self-healing fluorophores (e.g., trisNTA-Alexa647, Figure 3f).^134^ The advancements described above enhance the photostability of next-generation fluorophores, extend photobleaching time, and increase photon budgets, thereby advancing single-molecule biophysics studies.

### 3.3 Single-Molecule Fluorophore Photoswitchability

Photoswitchable fluorophores provide control over emissive and nonemissive states on time scales optimized for sequential localization of individual molecules. This property enables single molecule to ‘blink’ multiple times, a critical feature for overcoming the diffraction limit in densely labeled samples and essential for single-molecule localization-based super-resolution microscopy imaging.^30,32,145,146^ The cyanine dye family, exemplified by sulfo-Cy5 and Alexa Fluor 647, was introduced by the Zhuang lab as the first generation of photoswitchable fluorophores for super-resolution imaging when used in combination with switching buffer agents.^64–66^ Cyanine switching typically requires a carefully optimized photoswitching buffer of a thiol compound (e.g., mercaptoethylamine MEA),^65^ or phosphine tris­(2-carboxyethyl)­phosphine (TCEP),^63^ along with an enzymatic oxygen scavenging system to prevent photobleaching. Several research groups, including Sauer et al., Heilemann et al., and Cosa et al.,^29,54,147–149^ conducted additional experimental and theoretical investigations to further elaborate on the single-molecule photoswitching mechanism. Certain oxazine fluorophore Atto 655, have demonstrated effective photoswitching in the presence of oxygen using a redox buffer composed of methyl viologen (MV) and ascorbic acid (AA), as shown by the Tinnefeld group.^88^ Notably, Atto dyes require lower thiol concentrations for switching compared to cyanine dyes. Remarkably, naturally occurring glutathione present in cells enable synthetic Atto 655 derivations to be directly applied in live-cell STORM imaging in the presence of oxygen.^89^

Recent advancements have focused on fluorophores with spontaneously blinking (Figure 4a–h) or environmentally sensitive photoswitching (Figure 4i–n) properties, eliminating the need for buffer additives and enhancing compatibility with live-cell imaging. A typical example is the development of HMSiR dyes by the Urano group,^77^ which introduced the concept of spontaneously blinking fluorophores driven by intramolecular spirocyclization. Further improvements in these fluorophores have optimized key parameters such as rapid switching speeds, fast ON time, low ON/OFF ratio, and appropriate duty cycles, all of which are critical for the performance of single-molecule super-resolution microscopy techniques and their compatibility with multicolor imaging.^150^ These photoswitching properties of developed organic fluorophores and their applications in single-molecule localization-based super-resolution microscopy have been summarized in the literature.^29,90,146,151,152^ In 2023, Liu et al. reviewed advancements in spontaneously blinking rhodamines, emphasizing intramolecular spirocyclization mechanisms,^117^ while Kaur et al. discussed photochemical mechanisms of photoswitchable fluorophores and their modifications to enhance switching properties.^118^

**Figure 4:**
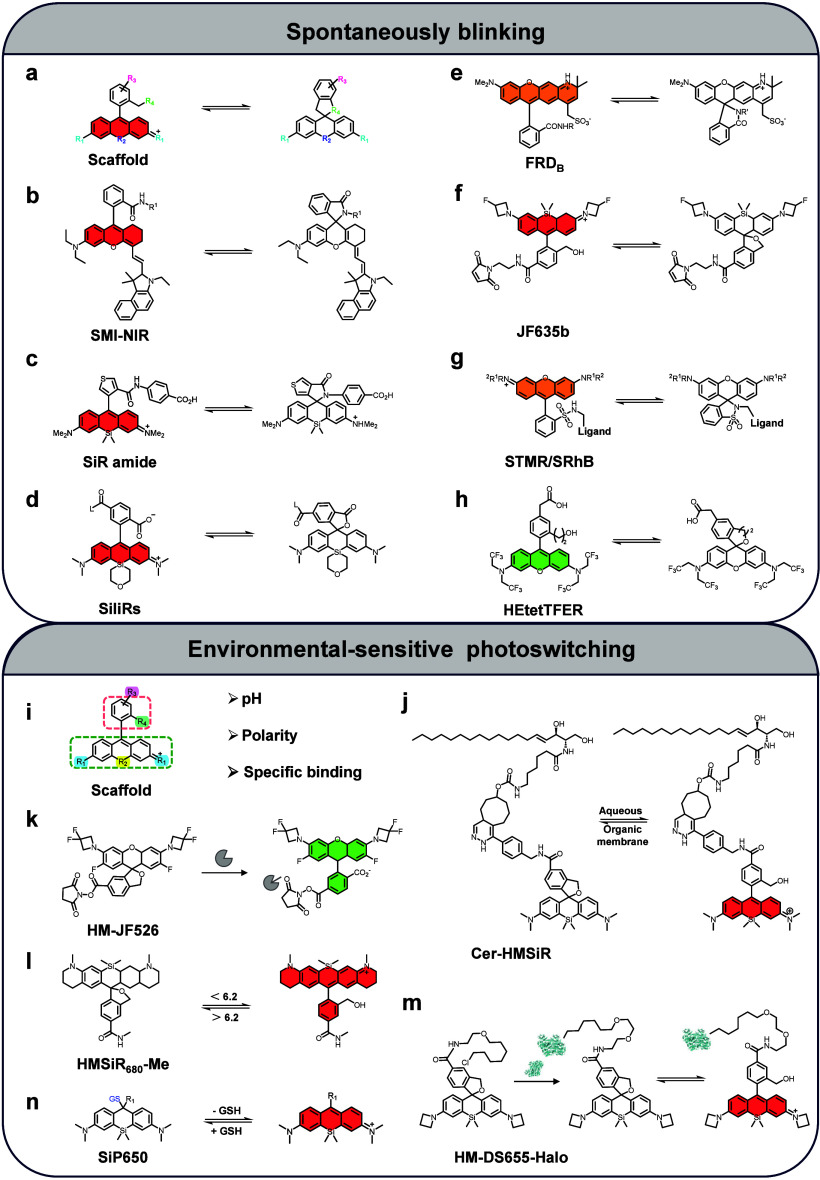
Next-generation fluorophore engineered for photoswitching in single-molecule localization-based super-resolution microscopy. (a) Core fluorophore structure - The fundamental molecular scaffold based on ring-opening rhodamine dyes with modifiable groups to enable photoswitching functionality. (b–h) Spontaneously blinking fluorophores - Fluorophores capable of stochastic transitions between fluorescent “on” and nonfluorescent “off” states without the need for external activators. (i–n) Environmentally sensitive photoswitching fluorophores: (i) molecular scaffold and (j–n) fluorogenic probes whose photoswitching behavior is influenced by biotarget environmental factors, including bilayer membrane polarity (j), specific protein–ligand interactions, such as living cells expressing the target protein fused to a HaloTag (k, m), and pH (l). The colors within the fluorophore structures indicate the positions of the ON states and correspond to their respective emission wavelengths.

Spontaneously blinking fluorophores have undergone significant advancements through strategic engineering of the Si-xanthene framework with distinct substitutions to modulate the spirocyclic state.^153^ Notable examples include the 721 nm near-infrared (NIR) illuminated SMI-NIR,^154^ the fast-blinking SiR amide with on-times of less than 3.0 ms,^155^ silinanyl rhodamines with low ON/OFF ratios,^156^ the rhodamine-derived fluorophore FRD-B incorporating a secondary amide group at the carboxyphenyl position of the rhodamine core,^157^ a series of hydroxymethyl-Si-rhodamine analogs (JF_635_b) with an intermediate duty cycle,^75^ self-blinking sulfonamide derivatives STMR and SRhB,^158^ and SiR-based

HMSiR (red-emitting) and HEtetTFER (green-emitting) (Figure 4b–h).^159^ In addition to these designs, fluorophores with environmentally responsive photoswitching properties have predominantly utilized the rhodamine scaffold. These fluorophores exhibit fluorescence switching triggered by specific environmental factors such as acidic organelle,^160^ reversible binding specifically to cell plasma membranes,^161,162^ or protein–ligand biomolecular interactions^163,164^ (Figure 4i–m). A noteworthy example is the xanthene fluorophore SiP650, developed by Morozumi et al. in 2020.^165^ This fluorophore achieves spontaneous blinking through an intracellular glutathione (GSH)-triggered reversible ground-state nucleophilic attack at the ninth carbon of the xanthene ring, offering a unique mechanism for photoswitching modulation (Figure 4n).^165^ More recently, the Rivera-Fuentes lab (2024) developed improved cyclization cyanine dyes that undergo more favorable exo-trig cyclization, enabling efficient switching for general SMLM imaging.^166^ Based on these investigations, fluorophores engineered for photoswitching, driven by innovative molecular designs, have emerged in recent years as a dominant focus for advancing super-resolution imaging.

Beyond experimental validation, significant progress has been made in the theoretical prediction of fluorophores’ blinking properties using physical chemistry models. Urano et al. (2018) and Schepartz et al. (2021) established a critical p*K*_cycling_ threshold (<6.0) necessary for effective blinking under physiological conditions.^76,159^ Liu and Xu et al. refined the criteria to a narrow p*K*_cycling_ window (5.3–6.0)^153^ and a Gibbs free energy range (*ΔG*_c‑o_ = 1.156–1.248 eV)^167^ as important parameters for designing self-blinking rhodamines with precise thermal equilibrium. In 2023, Xiao et al. identified the recruiting rate (*k*_rc_ = 0.1–5.0 s^–1^) as the temporal requirement for self-blinking rhodamines in live-cell super-resolution imaging.^168^ These theoretical contributions establish a robust framework for the rational design of photoswitching fluorophores for single-molecule localization-based super-resolution imaging.

### 3.4 Fluorophores with Photoactivatable Properties

Photoactivatable fluorophores are specialized single-molecule probes that typically transition irreversibly from a nonemissive (“off”) state to a fluorescent (“on”) state when exposed to specific wavelengths of light.^169^ These properties make them ideal for photoactivated localization microscopy (PALM) super-resolution imaging.^170^ Once activated, the on-state fluorophores must undergo photobleaching before new fluorophores are activated, allowing sequential activation of new fluorophores and ensuring precise temporal control over localization events in PALM. Achieving this functionality relies on a key structural modification that enables the fluorophore to switch states in response to light exposure, optimizing their performance in super-resolution imaging.^171^

Synthetic photoactivatable fluorophores can be broadly categorized into four classes, with the most extensively studied being photocaged fluorophores (Figure 5a–g). This category encompass numerous examples, including azetidinyl rhodamine derivatives (PA-JF_549_ and PA-JF_646_) introduced in 2016,^73^ nitroveratryl oxycarbonyl (NVOC)-caged Si-rhodamin in 2016,^172^ Spiropyran in 2020,^173^ caged rhodamine fluorophores modified with a photocleavable hydrophobic dimethoxy-2-nitrobenzyl group in 2012 ^174^ or hydrophilic SO_3_H groups in 2021,^175^ and photocaged meso-methyl BODIPYs in 2023.^176^ More recent examples include nitroso-caged rhodamine derivatives^177,178^ and nitroso-caged sulfonamide rhodamine (NOSR) probes developed in 2024.^179^

**Figure 5:**
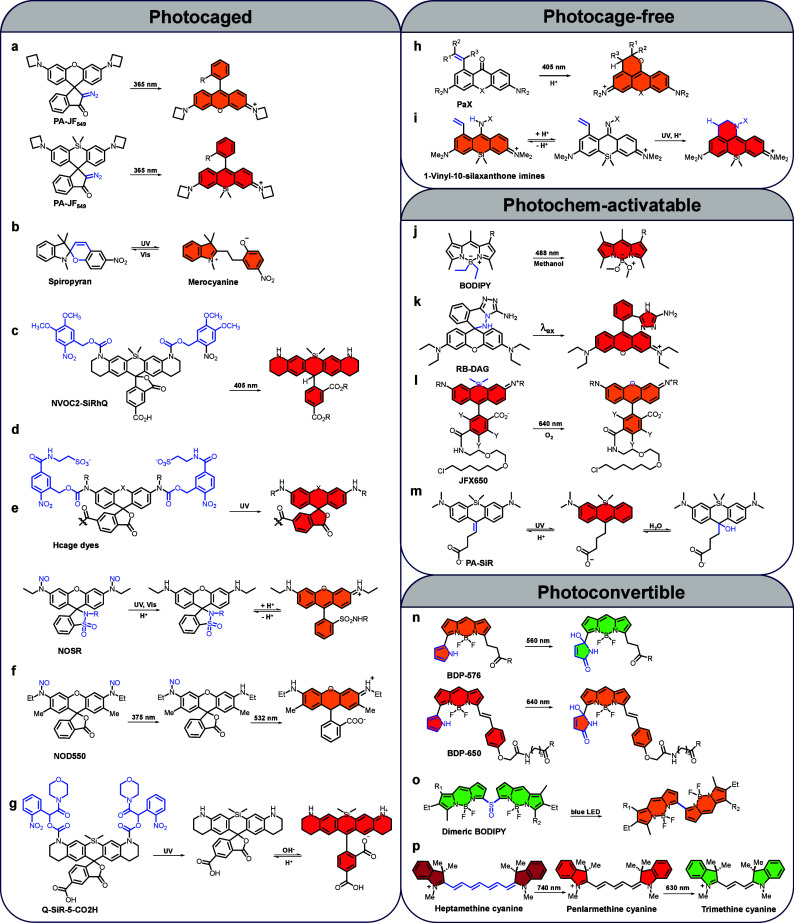
Representative fluorophore structures developed for photoactivated localization microscopy imaging. (a–g) Photocaged fluorophores - Fluorophores protected by photocleavable groups, which release active fluorescent species upon UV or visible light irradiation. (h, (i) Photocage-free fluorophores - Chemically engineered fluorophores featuring a 3,6-diaminoxanthone scaffold with an intramolecular alkene radical trap. (j–m) Photochem-activatable fluorophores - Fluorophores requiring both light exposure and chemical triggers to transition from a dark state to a fluorescent state. (n–p) Photoconvertible fluorophores - Fluorophores capable of undergoing irreversible spectral shifts upon light exposure. The blue color indicates the photoactivatable chemical moieties, while the colors within the fluorophore rings denote the positions of the ON states of the fluorophores and correspond to their respective emission wavelengths.

In contrast to photocaged fluorophores, the second category comprises photocage-free fluorophores (Figure 5h,i), a relatively recent development proposed within the past two years. Notably, Hell and colleagues introduced PaX fluorophores,^180,181^ which are based on a 3,6-diaminoxanthone scaffold incorporating an intramolecular alkene radical trap. These fluorophores undergo light-induced radical cyclization, resulting in highly photostable and fluorescent pyronine dyes with exceptional photostability and compatibility with MINFLUX nanoscopy.^84^ The Kikuchi group further employed PaX dyes to achieve spatiotemporal manipulation of gene expression.^85^ The third category includes photochem-activatable fluorophores (Figure 5j–m),^182^ which are characterized by their photoinduced chemical reactions with oxygen (O_2_),^183^ methanol,^184^ and hydrogen ions (H^+^).^185^ The last category is photoconvertible fluorophores (Figure 5n–p), which feature a change in the emission wavelength upon photoactivation. For instance, the rhodamine derivative JFX_669_ transitions from its conventional far-red emission, excited at 640 nm, to a red single-molecule signal that can be excited at 561 nm,^90^ which is significant as it enables PALM experiments without the need for UV light. Another notable example includes cyanine fluorophores, which undergo a characteristic photoconvertible reaction under light exposure.^186^ This process involves a transition from heptamethine cyanine (far-red emission) to pentamethine cyanine (red emission), and finally to trimethine cyanine (green emission), mediated by singlet oxygen and proceeding through a multistep mechanism. A key intermediate in this pathway is hydroperoxycyclobutanol, which forms initially and undergoes molecular rearrangements, resulting in the progressive shortening of the polymethine chain and corresponding shifts in emission wavelength. Further advancements include the development of photoconvertible pyrrolyl-BODIPYs by the Collot group,^187^ dimeric BODIPY fluorophores by the Hao group^188^ and far-red photoactivatable BODIPY dyes by the Raymo group,^189,190^ broadening the utility of fluorophores for photoactivatable super-resolution imaging applications. These structural modifications, as described above, enable the fluorophore to instantly switch from a nonfluorescent or longer-wavelength emission state to a fluorescent or shorter-wavelength emission state upon light activation. This advancement enhances temporal control over fluorescence “on” state, thereby improving the performance of fluorophores in localization-based super-resolution imaging.

## 4 SITE-SPECIFIC BIOLABELING STRATEGIES FOR SINGLE-MOLECULE BIOPHYSICS

Achieving site-specific labeling of biomolecules for single-molecule fluorescence studies facilitates the monitoring of conformational changes and local interactions in targeted regions.^191^ A well-established method for visualizing cellular biomolecules involves the genetically encoded fusion of the target of interest with fluorescent proteins.^192,193^ However, this approach falls outside the scope of this review, as advanced single-molecule measurements require fluorophores with greater stability and brightness. While organic fluorophores offer superior brightness and photostability compared to fluorescent proteins, their primary limitation is the lack of genetic encoding, necessitating additional steps such as chemical coupling reactions for site-specific attachment. Comprehensive reviews and primers have summarized advancements in homogeneous labeling methods for proteins,^194–199^ nucleic acids (DNA and RNA),^40,200,201^ and their complexes.^202,203^ In this section, we highlight emerging site-specific labeling technologies for proteins, nucleic acids, and RNPs in single-molecule biophysical studies, with a focus on advancements in noncovalent affinity labeling, click chemistry, fluorescent RNA aptamers, smFISH, and DNA-PAINT.

### 4.1 Strategies for Site-Specific Labeling of Proteins

For recombinant proteins expressed in *E. coli*, site-specific labeling with fluorophores is commonly achieved by targeting cysteine or amine groups, with cysteine residues being particularly advantageous due to their low abundance and reactive sulfhydryl (-SH) group.^196^ Maleimide-functionalized fluorophores are frequently employed to form covalent bonds with sulfhydryl groups under mild conditions.^204^ In such cases, fluorophores are introduced through site-specific labeling at engineered cysteine residues. Unreacted fluorophores must be rigorously removed to minimize background fluorescence and enhance signal clarity during single-molecule imaging. Beyond cysteine-based labeling, several advanced strategies have been developed to incorporate next-generation fluorophores in single-molecule biophysics applications.

#### 4.1.1 Affinity-Based Noncovalent Chelation Strategies

Site-specific labeling through noncovalent chelation employs amino acid-conjugated peptide motifs, such as tetracysteine,^205^ tetraserine,^206^ aspartate,^207^ and multihistidine (His_n_),^208^ to achieve high-affinity interactions with metal ion-coordinated fluorophores (Figure 6a). These motifs offer a minimalistic approach to protein labeling, without requiring complex genetic modifications or large protein fusions. Among these, the multihistidine motif (e.g., His_6_ or His_10_) was originally developed for protein purification due to its high affinity for transition metal complexes, typically by Ni­(II):nitrilotriacetic acid (Ni­(II):NTA).^209^ Building on this principle, the Hamachi group in 2021 utilized the His-tag/Ni^2+^-NTA interaction combined with an N-acyl-*N*-alkyl sulfonamide (NASA) reactive group to achieve selective labeling of lysine residues in proximity to the His-tag sequence.^210^ In 2018, the Tampé group introduced multivalent NTA complexes, including *bis*-, *tris*-, and *hexa*-NTA, to improve binding stability with His-tagged proteins.^211^ Notably, *tris*-NTA exhibited exceptionally high affinity (*K*_D_ = 0.1 nM) for His_10_-tagged proteins by leveraging strong interactions with Ni^2+^ ions. Structural optimizations, including the incorporation of cyclic and dendritic scaffolds, further enhanced both affinity and selectivity. By conjugating *hexa-*NTA to Alexa Fluor 647 fluorophores, they established a robust and highly specific fluorescence labeling strategy, optimized for single-molecule and super-resolution microscopy applications.^212^

**Figure 6:**
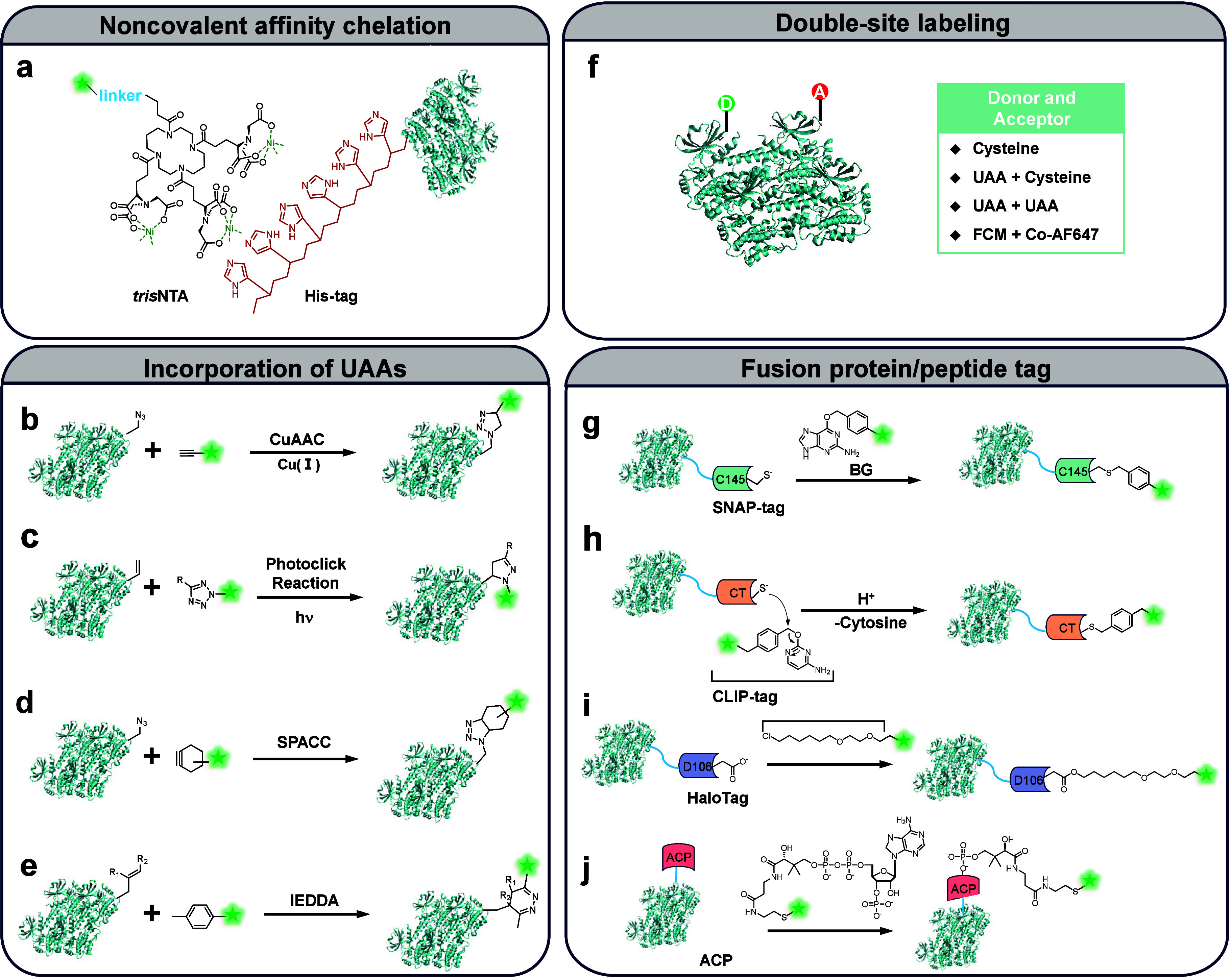
Advanced strategies for protein labeling using next-generation fluorophores in single-molecule fluorescence studies. (a) Noncovalent affinity chelation - Labeling strategies utilizing specific interaction between His-tag and Ni­(II)-trisNTA-fluorophore complexes. (b–e) Incorporation of unnatural amino acids (UAAs) - Strategies leveraging bioorthogonal reactions, including CuAAC (Copper-Catalyzed Azide–Alkyne Cycloaddition), SPAAC (Strain-Promoted Azide–Alkyne Cycloaddition), IEDDA (Inverse Electron Demand Diels–Alder), and photoclick reactions. (f) Double-site labeling for smFRET - Strategies employing combinations of labeling strategies, including double mutant cysteines, one UAA and one cysteine, two UAAs, or combinations such as one FCM (four-cysteine motif) and one Co-AF647 (coenzyme A conjugated fluorophore). (g–j) Fusion protein/peptide tags - Labeling techniques based on genetically encoded tags such as SNAP, CLIP, Halo, and ACP tags, allowing covalent attachment of fluorophores through ligand conjugation.

#### 4.1.2 Unnatural Amino Acid Incorporation Strategies

Unnatural amino acids (UAAs) can be introduced into proteins either *in vitro* through synthetic protein production,^213^ or *in vivo* by utilizing engineered organisms equipped with the necessary molecular machinery by using the Amber codon suppression strategy.^214^ The *in vivo* approach is typically genetically encoded by employing an orthogonal tRNA and aminoacyl-tRNA synthetase pair.^215^ This method enables the site-specific incorporation of UAAs containing functional groups such as ketone, azide, strained alkyne, or alkene, into the target protein that can be selectively labeled with fluorophores through bio-orthogonal reactions.^216^ Among these, azide-bearing UAAs are particularly favored due to their compatibility with copper-catalyzed azide–alkyne cycloaddition (CuAAC) click chemistry.^38^ In addition to CuAAC, other bio-orthogonal reactions employed for site-specific labeling include strain-promoted azide–alkyne cycloaddition (SPAAC), inverse electron-demand Diels–Alder (IEDDA) reactions, and photoclick chemistry (Figure 6b–e).^217–219^ In addition, non-natural fluorescent amino acids (FlAAs), which inherently possess fluorescent properties and do not require secondary conjugation, have emerged as powerful tools for protein labeling.^220^ Examples include BODIPY amino acid,^221^ 4-cyanotryptophan,^222^ and dansyl alanine,^223^ which offer unique optical properties such as environmental sensitivity, metal chelation responsiveness, tunable fluorescence, and prolonged fluorescence lifetimes. These FlAAs can be site-specifically incorporated into proteins via solid-phase peptide synthesis or genetic encoding, enabling the investigation of protein conformational changes and interactions in live cells.^224^

#### 4.1.3 Fusion Protein/Peptide Tagging Strategies

Effective single-molecule labeling using this strategy requires tags that are both small and minimally disruptive to the protein’s structure and function. Strong and specific conjugation between the fluorophores and the tag is typically achieved through enzyme-catalyzed covalent bond formation (referred to as enzyme self-labeling^225^) or via click chemistry,^197^ ensuring robust and reliable fluorophore labeling.

SNAP-tag,^226^ CLIP-tag,^227^ HaloTag,^228^ and ACP (acyl carrier protein)-derived tags^229–231^ are widely employed in single-molecule imaging for investigating dynamic cellular processes with minimal disturbance to the native protein environment (Figure 6g–j).^232^ By expressing the protein of interest fused to one of these small tags and labeling it with a cell-permeable, ligand-functionalized fluorophores, these systems enable reliable and long-term tracking of single molecules within living cells. Importantly, SNAP-tag, CLIP-tag, and HaloTag are not limited to recombinant proteins expressed in *E. coli*; they exhibit significant versatility in protein labeling across various expression systems, including mammalian cells, yeast, and insect cells.^233^ SNAP-tag and CLIP-tag, derived from O6-alkylguanine-DNA alkyltransferase (AGT), react with benzylguanine (BG) and benzylcytosine (BC) derivatives, respectively.^227^ HaloTag, based on haloalkane dehalogenase, covalently binds to haloalkane-functionalized substrates.^234^ These tags are not inherently fluorescent and require conjugation to fluorescent dyes. In contrast, ACP tags utilize enzymatic transfer of CoA-conjugated fluorophores to a specific serine residue but are limited to cell membrane labeling due to CoA’s lack of cell permeability.^235^

Site-specific protein labeling systems, such as SNAP-tag, CLIP-tag, and HaloTag, have revolutionized the application of next-generation fluorophores in live-cell fluorescence microscopy and super-resolution imaging. Recent advancements highlight the development of innovative ligands and fluorophores tailored to enhance labeling performance and imaging outcomes. Kompa et al. (2023)^236^ and Catapano et al. (2024)^237^ introduced exchangeable HaloTag ligands and the self-labeling protein tag HT7, both combined with fluorogenic silicon rhodamine derivatives. These innovations enable single-molecule tracking for durations exceeding 30 min and facilitate super-resolution microscopy with improved temporal resolution. Additionally, Janelia Fluor dyes, JF_549_ and JF_646_, conjugated to HaloTag and SNAP-tag ligands, exhibit high brightness, exceptional photostability, and cell permeability in single-molecule tracking and super-resolution imaging.^72^ Further studies identified Dy549 and CF640 as optimal fluorophores for SNAP-tag labeling in live-cell imaging due to their minimal photobleaching and low nonspecific binding.^238^ Photoactivatable Janelia Fluor dyes have been specifically engineered for use with HaloTag and SNAP-tag labeling systems. These modifications enable controlled activation and significantly enhanced brightness, optimizing their application for localization-based super-resolution microscopy.^73^ Comparative analyses between HaloTag and SNAP-tag systems further highlight the superior performance of rhodamine derivatives when conjugated to HaloTag, exhibiting up to 9-fold higher signal intensity and making them especially advantageous for STED super-resolution imaging.^239^

#### 4.1.4 Dual-Site Labeling Strategies

Double-site labeling is typically essential for smFRET experiments to measure molecular distances and dynamic conformational changes. Beyond labeling site specificity, high labeling efficiency is particularly critical, as it directly influences the reliability and interpretability of multicolor single-molecule fluorescence experiments.^240^ Foundational work by Ha et al. and Swiss et al. pioneered smFRET techniques, leading to significant advancements in microscopy methods, fluorophore selection, optimized labeling strategies, refined calibration approaches, and diverse biophysical applications.^241,242^ Accurate FRET efficiency measurements and reliable experimental outcomes depend on site-specific labeling of donor and acceptor fluorophores (Figure 6f). The careful selection of mutation sites is critical to minimize local environmental effects that could alter fluorophore performance. Traditionally, double-cysteine mutants of the protein of interest are engineered to facilitate labeling with maleimide-conjugated fluorophores. In 2011, Seo et al. introduced a hybrid approach combining the incorporation of unnatural amino acids (UAAs) at specific sites with conventional cysteine labeling.^243,244^ Using maltose-binding protein (MBP) as a model system, their study demonstrated significant improvements in smFRET data quality compared to dual-cysteine labeling, resulting in clearer distinctions between folded and unfolded protein states. In an alternative approach, Fernandes et al. (2017) developed an orthogonal labeling strategy for smFRET using a peptide containing both a four-cysteine motif (FCM) and the ybbR tag.^245^ In this proof-of-concept study, the FCM was labeled with FlAsH, which specifically binds to the tetracysteine motif, while CoA-AF647 (Alexa Fluor 647 conjugated to Coenzyme A) was site-specifically attached to an active serine residue near the N-terminus via 4′-phosphopantetheinyl transferase. These double-site labeling strategies ensure precise FRET measurements while maintaining the functional integrity of the target protein.

### 4.2 Strategies for Site-Specific Labeling of Nucleic Acids

#### 4.2.1 Chemically Engineering Reactive Groups

The most widely used method for introducing reactive groups for fluorophores into nucleic acids is solid-phase synthesis of DNA or RNA strands, which is typically limited to lengths of approximately 100 bases. This method leverages phosphoramidite chemistry, enabling step-by-step incorporation of modified and protected nucleotides, allowing for precise site-specific modifications and fluorophore labeling. Modifications can be introduced at the sugar, base, or phosphate backbone of the nucleic acids. Additionally, a range of modifiable building blocks, including the nucleobases and the ribose moiety, are available, further enhancing the flexibility and precision of this labeling approach for advanced imaging and biophysical studies. In 2016, Egloff et al. demonstrated the utility of sequence-specific postsynthetic oligonucleotide labeling, which has become a cornerstone technique for single-molecule fluorescence studies.^246^ Their method involves introducing 12-alkyne-etheno-adenine modifications at target adenine sites via DNA-templated synthesis, followed by CuAAC click chemistry with azide-functionalized Cy3 fluorophores (Figure 7a). Similarly, the Freisinger group proposed a dual-labeling strategy targeting RNA adenine residues.^247^ This approach uses a custom DNA strand to direct a reactive group to a specific adenine, introducing an alkyne for Cy5-azide dye conjugation via CuAAC click reaction, while simultaneously oxidizing the 3′ ribose to a dialdehyde for Cy3-hydrazide attachment. These chemical coupling strategies introduce small, reactive groups that are compatible with subsequent reactions, such as RNA ligation, further underscoring their utility for single-molecule biophysics.

**Figure 7:**
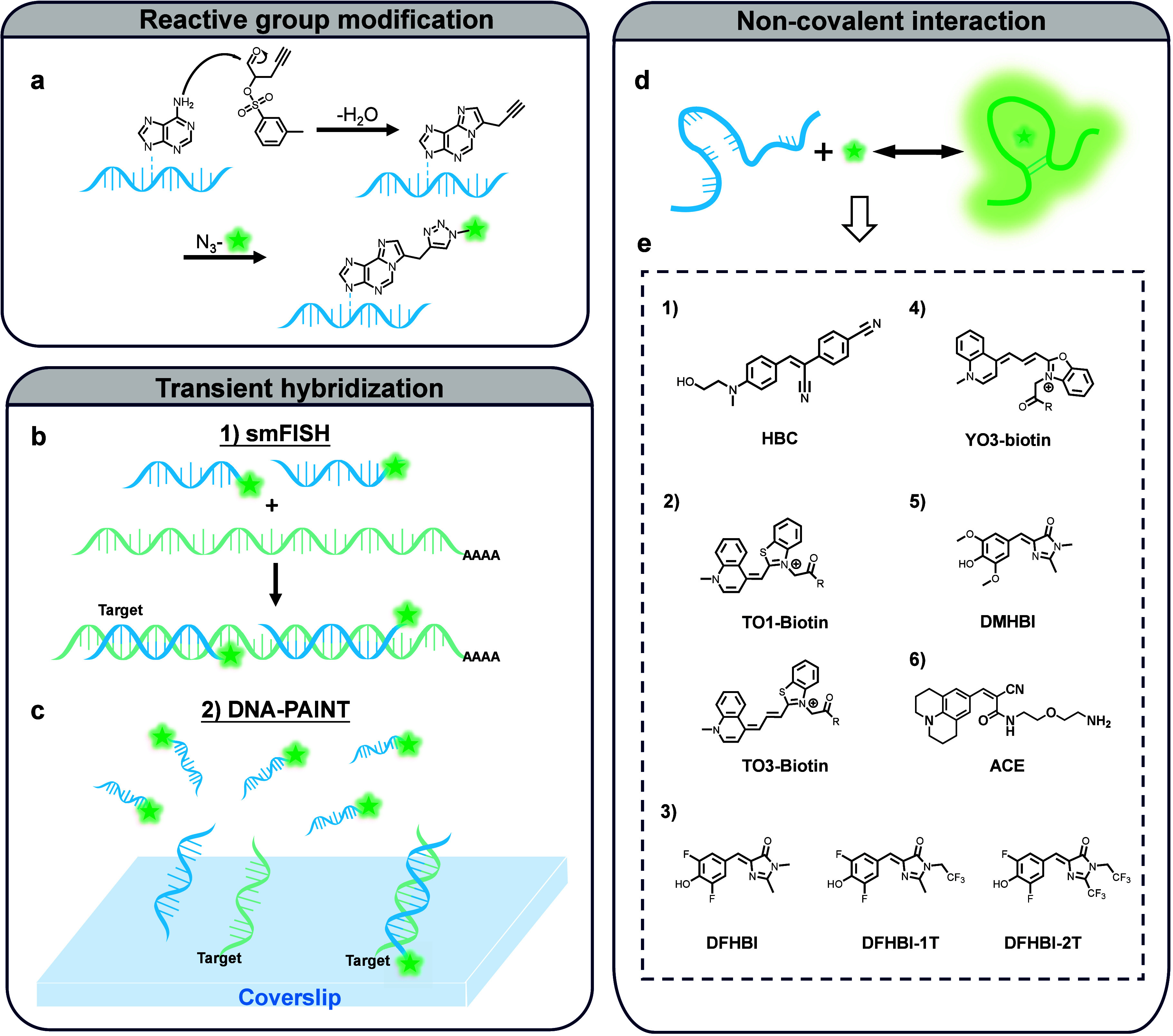
Advanced strategies for nucleic acid labeling using next-generation fluorophores in single-molecule biophysics. (a) Site-specific labeling of nucleic acids through chemically reactive modifications of bioorthogonal groups. (b, c) Transient hybridization-based labeling - approaches including smFISH (single-molecule fluorescence in situ hybridization) (b) and DNA-PAINT (point accumulation for imaging in nanoscale topography) (c) that rely on transient hybridization of complementary DNA probes for localization-based super-resolution imaging. (d) Labeling based on the reversible binding of fluorophores to nucleic acids through noncovalent interactions. (e) Structures of next-generation fluorophores specifically designed to bind nucleic acids noncovalently.

#### 4.2.2 Strategies Utilizing Transient Hybridization

Transient hybridization-based labeling methods (Figure 7b,c), which do not require chemical modification of nucleic acids, employ complementary DNA sequence probes conjugated with fluorescent tags to selectively bind to target DNA or RNA sequences.^30^ Among these approaches, single-molecule fluorescence in situ hybridization (smFISH)^248^ is widely used for the localization of individual mRNAs in fixed cells. This technique utilizes multiple short, fluorescently labeled oligonucleotide probes that hybridize to distinct regions of a target RNA (Figure 7b). Raj et al. advanced smFISH by introducing a method with 48 independent probes, each approximately 20 bp in length, conjugated to fluorescent dyes.^249^ This approach simplified probe synthesis while enhancing signal strength and enabling precise transcript localization and mRNA quantification. Recent developments have extended the capabilities of smFISH for high-throughput multiplexed RNA detection. Techniques such as SeqFISH^250^ and MERFISH^251^ enable the simultaneous detection of thousands of RNA species, while ClampFISH^252^ enhances probe stability and specificity through click chemistry. Additionally, smiFISH,^253^ a cost-effective variation, employs unlabeled primary probes paired with fluorescently labeled secondary detector probes. This strategy increases flexibility and facilitates multicolor labeling without requiring new probe synthesis. As a result, smFISH and its derivatives have become indispensable tools for investigating RNAs in fixed cells.

PAINT (Points Accumulation for Imaging in Nanoscale Topography) is an alternative single-molecule localization microscopy technique that employs transient hybridization or interaction between target molecules and freely diffusing dyes or dye-labeled ligands.^254^ DNA-PAINT, introduced by Jungmann et al. in 2010,^255^ employs complementary DNA hybridization to achieve transient binding of fluorescently labeled probes to target molecules (Figure 7c).^256,257^ In this method, a docking strand immobilized on the biological sample hybridizes with an imager strand conjugated to a fluorophore that diffuses freely in solution. The imager strand is covalently conjugated with fluorophores including the optimal dyes Cy3B and Atto 655, identified through a comprehensive performance analysis of fluorescent dyes.^87^ For optimal fluorescence signal detection under microscopy, the fluorophore is strategically positioned at the terminus of the imager strand. A secondary-label-based DNA-PAINT approach has recently been developed, enabling highly multiplexed imaging with single-protein resolution.^258^ In this method, target proteins were initially labeled with a primary DNA barcode. A secondary label was subsequently introduced, comprising three key components: the full complement sequence of the primary barcode, a toehold region to facilitate hybridization, and speed-optimized DNA-PAINT docking sequences to enhance imaging efficiency. Upon complementary binding, the imager strand remains stationary long enough to allow sufficient photon collection for accurate single-molecule localization. The reversible nature of this binding facilitates repeated interactions, thereby increasing photon budgets for super-resolution imaging studies. By employing uniquely designed DNA sequences and distinct fluorophores, DNA-PAINT enables simultaneous visualization of multiple targets, with each target associated with a specific color or sequence code, thus facilitating super-resolution multiplexed imaging.^52^

#### 4.2.3 Strategies Leveraging Noncovalent Intermolecular Interactions

A minimally invasive labeling strategy for nucleic acids leverages noncovalent interactions such as hydrogen bonding, π-π stacking, and van der Waals forces to stabilize fluorophores in fluorescent conformations (Figure 7d). For example, DFHBI, a GFP chromophore analog, intercalates between DNA base pairs, stabilizing its fluorescence.^259^ Similarly, SYTOX^260^ and YO^261^ derivatives are widely favored due to their fluorescence enhancement—up to 1000-fold—upon DNA binding, offering the high sensitivity required for single-molecule imaging. RNA aptamers utilize this mechanism to enhance fluorescence signals upon binding specific fluorophores, enabling real-time, noninvasive monitoring of RNA localization, expression, and dynamics in live cells.^262^ The aptamer creates a precise binding pocket for the fluorophore, restricting molecular motion, preventing nonradiative decay, and shielding it from solvent quenching, thereby enhancing fluorescence.^41^ These features make fluorescent RNA aptamers highly effective tools for single-molecule studies of RNA in living cells.

Fluorescent RNA aptamers, first introduced by Jaffrey et al. in 2011,^263^ have since evolved with notable examples including Spinach, Broccoli,^264^ Mango,^265^ and Corn^266^ in the last years. Recent advances focus on improving fluorescence performance for real-time RNA imaging. For instance, the Yang group developed synthetic dyes HBC^82^ and ACE^267^ (Figure 7e), paired with the D11 aptamer, which boosts HBC fluorescence over 3,000-fold by preventing nonradiative decay, enabling RNA tracking in *E. coli*. They also introduced the Okra aptamer,^267^ optimized for brightness, low ion-dependence, and multicolor super-resolution imaging. Additionally, the Clivia aptamer,^83^ which binds to the NBSI dye, features a large Stokes shift (250–300 nm), making it ideal for imaging in autofluorescent or spectrally complex environments. Separately, Wirth et al. 2019, reported that the SiRA aptamer stabilizes the zwitterionic fluorescent form of silicon rhodamines, achieving near-infrared emission, exceptional brightness, and compatibility with live-cell and super-resolution microscopy.^268^ These aptamers bind specifically to small-molecule fluorophores, with each interaction displaying unique fluorescence characteristics and offering distinct biophysical applications.

### 4.3 Labeling Strategies for RNA–Protein Complexes

RNA-protein complexes (RNPs), including ribosomes, spliceosomes, signal recognition particles, and RNA-induced silencing complexes, are essential molecular machines involved in gene expression and protein synthesis, whose dynamic conformational changes and transient intermediates can be investigated in real time using single-molecule spectroscopy and microscopy techniques.^269–271^ Site-specific labeling techniques have emerged as basic tools for investigating the structural dynamics and interactions of RNPs in single-molecule studies. These strategies, similarly adapted from protein and RNA labeling methods discussed in the previous section, utilize cysteine residues, unnatural amino acids, protein tags, click chemistry reactions, as well as in situ hybridization, to enable the precise attachment of fluorophores to specific RNPs.^272,273^ Dual-color labeling of RNP subunits and associated factors has proven to be a powerful approach for supporting smFRET analysis of their dynamic behavior. A notable example, in 2021 Rundlet et al. investigated early translocation events in the ribosome, where the subunits uS13 and uL1 were site-specifically labeled with donor (LD550) and acceptor (LD650) fluorophores via cysteine-maleimide conjugation, meanwhile, mRNA molecules are biotinylated and immobilized on passivated surfaces through streptavidin, ensuring stable orientation of ribosomal complexes for observation.^274^ Their findings suggest that elongation factor G engages pretranslocation ribosome complexes in an active and GTP-bound conformation to initiate the unlocking of peptidyl-tRNA. In another study examining the ribosomal mechanism for signal sequence handover, single-molecule fluorescence analysis involved precise labeling of ribosomal proteins, such as uS19 and uL18, at their N- and C-termini, respectively, using the fluorophores Cy3 and Cy5.^275^ This labeling strategy enabled real-time smFRET tracking of tRNA positioning and interactions during translocation, providing critical insights into ribosomal dynamics and conformational transitions.

## 5 UTILIZING NEXT-GENERATION FLUOROPHORES FOR SINGLE-MOLECULE STUDIES

Recent advancements in next-generation fluorophores and their labeling methodologies have significantly enhanced our understanding of the single-molecule dynamics of membrane transporters and proteins,^276,277^ including superfamilies like ion channels,^278^ ATP-binding cassette (ABC) transporters,^279^ G protein-coupled receptors (GPCRs),^11–14^ receptor tyrosine kinases (RTKs) and other transmembrane receptors,^280–284^ phase-separating proteins,^285^ and CRISPR-associated endonuclease.^286–288^ These innovations have shed light on fundamental processes such as substrate transport, ion flux, signal transduction, phosphorylation, and ATP synthesis, providing critical insights that inform drug design and elucidate the mechanisms underlying biomolecular function.^289–291^ Fluorescence microscopy techniques including single-molecule fluorescence resonance energy transfer (smFRET) and single-molecule localization-based super-resolution microscopy, have been instrumental in probing these dynamic processes through the use of labeled fluorophores.^241,292^ This section highlights how next-generation fluorophores and their tailored labeling strategies contribute to improved temporal and spatial resolution in single-molecule biophysics.

### 5.1 Improving Temporal Resolution to Observe Fast Dynamics

Highly photostable and bright fluorophores play a pivotal role in single-molecule fluorescence studies of protein dynamics, particularly in smFRET measurements. Their exceptional photophysical properties enable precise monitoring of protein dynamic events by minimizing signal loss caused by photobleaching or unwanted blinking. This, in turn, ensures reliable and consistent data acquisition, leading to improved signal-to-noise ratios and enhanced data fidelity. Nevertheless, despite these advancements, significant challenges persist in capturing fast conformational fluctuations within the submillisecond time regime or analyzing transition path times. These limitations arise primarily from the requirement for MHz photon count rates of labeled fluorephores,^293–295^ a benchmark that current techniques struggle to achieve.

“Self-healing” fluorophores, such as the LD555 or LD555p (donor) and LD655 (acceptor) FRET pair, derived from sulfo-Cy5 fluorophores modified with cyclooctatetraene (COT), can reduce triplet-state accumulation without requiring additional additives, thereby enhancing the temporal resolution limits of smFRET and enabling more precise observation of dynamic biomolecular processes.^92^ Using this, the Blanchard lab examined secondary active MhsT transporter at the single-molecule level.^91^ In this study, a ligand-binding-protein-scaffold (LIV-BP) sensor labeled with FRET dye pairs (LD555p and LD655) has been utilized to achieve an enhanced temporal resolution in smFRET traces, reducing it to below 1.0 ms. This improvement allowed for the observation of the LIV-BPSS sensor exhibiting two distinctly defined low- and high-FRET states (Figure 8, left). By reducing triplet-state accumulation and increasing the photon budget, smFRET traces exhibit smoother and more distinct state transitions. This is particularly critical for single-molecule transport studies, where subtle variations in FRET efficiency correspond to molecular conformational changes or substrate interactions. These enhancements enable smFRET imaging to capture rapid transitions in the protein conformational cycle, as reflected in the dwell-time distributions of different FRET states. Furthermore, high-temporal-resolution imaging of single-molecule fluorescence enabled more accurate detection of transitions between low- and high-FRET states at millisecond-scale resolution. Furthermore, single-turnover measurements indicated that the transport rate of MhsT for leucine ligand was approximately 0.62 ± 0.08 s^–1^ by using the LIV-BP sensor under specific buffer conditions. Using the similar self-healing FRET pairs in combination with molecular dynamics simulations, Girodat et al. 2020, achieved time resolutions of even 0.25 ms, enabling the detection of rapid conformational changes in the leucine/isoleucine/valine-binding protein from *E. coli*.^296^ In another significant study, Morse et al. 2020, employed three-color smFRET to investigate the conformational dynamics of Elongation Factor Tu (EF-Tu) and aminoacyl-tRNA during proofreading on the ribosome.^96^ In this critical experiment, EF-Tu, tRNA, and ribosomal tRNA were labeled with LD750, LD650, and Cy3B, respectively, achieving a temporal resolution of 2.0 ms to observe rapid conformational changes. The dissociation of EF-Tu was indicated by the loss of the LD650-LD750 FRET signal, while a sharp increase in the LD650-Cy3B FRET indicated interactions between tRNA molecules. Furthermore, Gregorio et al. 2017, utilized optimized self-healing Cy3B and Cy7 FRET pairs to capture subtle conformational changes of the β2-adrenergic receptor induced by various ligands, demonstrating a Förster distance of approximately 5.0 nm and a temporal resolution of 100–500 transitions per second.^12^ Collectively, these self-healing fluorophores delivered improved temporal resolution for the observation of dynamic structural transitions.

**Figure 8:**
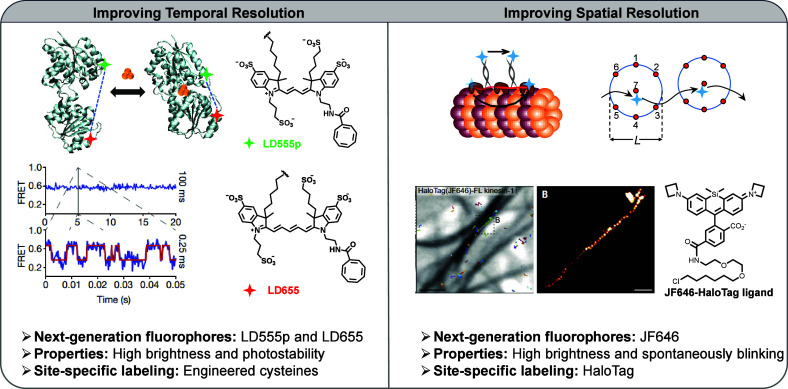
Representative advancements enabled by next-generation fluorophores and corresponding site-specific labeling techniques, illustrating their impact on enhancing temporal (left) and spatial (right) imaging resolution in single-molecule biophysics. Left, apo and substrate-bound structures of LIV-BP (leucine, isoleucine, valine periplasmic binding protein) labeled with LD555p and LD655 fluorophores. Single-molecule FRET (smFRET) traces of LIV-BP at 3.8 μM leucine ligand illustrating molecular dynamics captured at 100 ms and 0.25 ms time resolutions. The smFRET traces were reproduced with permission from ref 91. Right, MINFLUX microscopy achieves nanometer-level precision for tracking kinesin’s movement along microtubules. Kinesin moves along microtubules in a hand-overhand manner, with the apparent step size dependent upon the labeling position. This discrepancy arises from the spatial offset between the fluorophore and kinesin’s center of mass. A donut-shaped laser beam sequentially probes seven positions (1–7) surrounding the fluorophore to pinpoint its precise location. The scanning pattern dynamically recenters on the fluorophore, iteratively refining its position. The images were reproduced with permission from ref 74. Scale bars: 1 μm and 100 nm.

Bright Alexa Fluor (AF) dyes and Atto dyes have also proven to be robust tools for smFRET analysis of the multistate conformational dynamics of G protein-coupled receptors (GPCRs). For instance, Alexa Fluor 546 (donor) and Alexa Fluor 647 (acceptor) FRET fluorophores were employed to label metabotropic glutamate (mGlu) receptors using a live-cell-compatible click chemistry approach. This methodology enabled the investigation of dynamic conformational exchanges based on a three-state receptor activation model, encompassing the resting open–open (ROO), resting closed–closed (RCC), and active closed–closed (ACC) states.^94^ Similarly, in 2023, Maslov et al. utilized Alexa Fluor 488 and Atto 643, based on multiparameter fluorescence detection with pulsed interleaved excitation (MFD-PIE) smFRET, to examine the conformational dynamics (∼ 2.0 ms) of the A_2A_ adenosine receptor reconstituted in lipid nanodiscs, thus preserving a native-like membrane environment.^95^ Notably, it demonstrated the receptor rapid transitions, approximately 390 ± 80 μs, in the agonist-bound state. These high-performance fluorophores, when applied in GPCR studies, have significantly advanced our understanding of receptor activation and modulation, underscoring their potential for driving progress in drug discovery and mechanistic biophysics.

Significant progress in improving the temporal resolution of fluorophores for smFRET analysis has been achieved through the implementation of DNA origami nanoantennas with plasmonic hotspots.^297^ The nanoantennas enhance the local electric field surrounding fluorophores, improve photostability by reducing the time spent in reactive excited states, and boost photon emission rates.^298^ This approach has enabled the observation of protein–protein interactions with transient complex lifetimes of 100 μs and DNA hybridization events with transition times as short as 17 μs using the Cy5B/Dy-751 FRET pair.^93^ Complementing this strategy, the DyeCycling^299^ method addresses the major limitation of irreversible photobleaching, enabling dynamic fluorophore replacement to sustain fluorescence signals and extend experimental durations to several hours in smFRET without data loss.^300,301^ These advancements represent important approaches for enhancing the time resolution of next-generation fluorophores for the study of ultrafast biomolecular dynamics.

### 5.2 Improving Spatial Resolution for Super-Resolution Microscopy Imaging

Next-generation fluorophores have advanced super-resolution microscopy by improving signal quality and localization precision, primarily determined by their photon budgets. Fluorophores from the cyanine family including Alexa Fluor 647, CF647, CF660, and CF680, as well as those from the oxazine Atto family, exhibit high quantum yields, efficient photoswitching, and substantial photon budgets, thereby enhancing signal-to-noise ratios and enabling precise localization of nanoscale structures in dSTORM super resolution imaging. Martens et al. demonstrated the potential of CF and Atto fluorophores in achieving sub-20 nm resolution in super-resolution imaging of clathrin-coated pits and tubulin filaments.^68^ Meanwhile, the narrow spectral profiles of CF dyes facilitated multiplexed imaging through spectral demixing-based, registration-free multicolor dSTORM with minimal crosstalk.^302^ Sauer et al. explored the use of photoswitchable Alexa Fluor 647 fluorophores in super-resolution imaging, detailing their ability to transition between ON and OFF states and achieve lateral (10–20 nm) and axial (50–60 nm) resolution.^303^ Lehmann et al. optimized chemical caging strategies and screened highly bright Atto fluorophores, enabling the resolution of 40 nm synaptic vesicle ultrastructures in brain sections with minimal crosstalk and ∼ 20 nm localization precision in super-resolution imaging.^304^ Furthermore, Atto fluorophores were employed in DNA-PAINT, enabling simultaneous multicolor imaging without requiring sequential fluid exchange and achieving localization precision of 3–6 nm.^305^ Far-red fluorophores, particularly those containing cyanine 7 backbones and silicon rhodamines,^268^ outperform most dyes in other wavelength ranges in terms of the number of detected photons per switching event, on–off duty cycle, and number of switching cycles, which enhanced the signal-to-noise ratio and imaging resolution in single-molecule localization microscopy.^306^ Furthermore, fluorinated silicon-rhodamines and phosphorylated oxazines have been demonstrated as advanced fluorophores, achieving resolutions below 20 nm while offering enhanced photostability for STED super-resolution microscopy.^307^ Impressively, silicon-rhodamines, when combined with HaloTag system with exchangeable ligands (xHTLs),^308^ provided reversible, noncovalent binding with rapid kinetics (*k*_on_ > 10^6^ M^–1^ s^–1^, *k*_off_ ≈ 1 s^–1^), and achieved resolutions of approximately 4 nm in DNA-PAINT super-resolution imaging.^236^

The development of next-generation fluorophores is advancing in parallel with newly developed three-dimensional 4Pi-SMS^309^ (4Pi single-molecule switching) microscopy techniques,^310^ as well as super-resolution nanoscopy techniques, including MINSTED,^311^ RESI (resolution enhancement by sequential imaging),^312^ ROSE (radial interferometric single-molecule localization microscopy),^313^ ONE (one-nanometer expansion microscopy),^314^ and MINFLUX (minimal photon fluxes).^53,315^ Using the super-resolution MINFLUX technique, the Reis lab achieved nanometer-scale spatial resolution to track the stepping motion of the motor protein kinesin-1 along microtubules in both two and three dimensions (Figure 8, right).^74^ This was achieved by labeling with a HaloTag ligand conjugated to the highly bright and spontaneously blinking dye JF646, utilizing the principles of single-molecule tracking (SMT)^316–318^ in live cells. Specifically, the technique exploited the high brightness of JF_646_ using a donut-shaped excitation beam, which strategically positions fluorophores in the low-intensity ‘dark’ center, minimizing photobleaching and extending photon emission durations. The fluorophore was site-specifically attached to regions such as the C-terminal cargo-binding domain or the N-terminal motor domain via small protein tags, reducing linkage errors to ≈3 nm, comparable to the system’s resolution. This site-specific labeling strategy enables accurate interpretation of step sizes and motor protein dynamics while minimizing positional uncertainty. These improved fluorophore properties and biolabeling strategies have revealed previously unobservable zigzag trajectories of kinesin, providing a deeper understanding of molecular motor mechanics in their native cellular context. Additionally, photoactivatable carbo- and silicon-rhodamines with UV–vis-controlled activation have been structurally modified to enable nanometer resolution imaging with localization precision of 2.2–3.2 nm, facilitating superprecision 2D and 3D imaging of cellular structures such as nuclear pores and vimentin filaments.^319^ Moreover, the emerging MINSTED concept has demonstrated the ability to achieve localization precisions in the Ångström range at room temperature, leveraging blue-shifted STED microscopy to isolate individual fluorophores for applications such as imaging nuclear pore complexes and other cellular structures, further extending the spatial limits of super-resolution imaging techniques.^320^

The integration of physicochemical parameters, including fluorescence lifetime, polarization, and anisotropy properties of next-generation fluorophores, with single-molecule localization microscopy represents a promising advancement in super-resolution imaging.^321,322^ In 2024, the Xu group provided a comprehensive review on single-molecule spectroscopy and super-resolution mapping of physicochemical parameters in living cells.^322^ These unique fluorophore properties mitigate chromatic aberration and enable multiplexed imaging of multiple targets even with spectral overlap.^323^ Enderlein and colleagues demonstrated the benefits of leveraging cyanine-backbone fluorophores’ fluorescence lifetime properties in single-molecule super-resolution microscopy.^321,324,325^ Using fluorescence lifetime image scanning microscopy (FL-iSMLM), they achieved a lateral resolution of approximately 5.7 nm, nearly doubling the standard resolution.^324^ Additionally, combining fluorescence lifetime with metal-induced energy transfer (MIET) imaging provides isotropic nanometer-scale accuracy in three dimensions.^326^ These advancements illustrate that, beyond brightness parameters, fluorescence lifetime serves as a potential tool for enabling multiplexing capabilities in super-resolution microscopy.

## 6 CONCLUSIONS AND PERSPECTIVE

Recent advancements in next-generation organic fluorophores and their corresponding site-specific biolabeling techniques have markedly improved the temporal and spatial resolution of imaging in single-molecule biophysics. Fluorophores with enhanced photostability, brightness, photoswitching, and photoactivatable properties have enabled more reliable and extended single-molecule fluorescence tracking, as well as single-molecule localization-based super-resolution imaging of biological systems, driving breakthroughs in understanding dynamic biological processes. Simultaneously, precision in site-specific biolabeling has enabled researchers to monitor targets of interest in detail while preserving the native behavior of the molecules under observation. Together, these innovations are transforming our ability to investigate and understand molecular behavior at the single-molecule level.

Despite these advances, fully unlocking the potential of single-molecule fluorescence biophysics necessitates further advancements in fluorophore design and biolabeling strategies. Biological systems exhibit increasing levels of complexity, necessitating higher-brightness and photostable information content and prolonged imaging durations. However, these requirements are often constrained by unwanted fluctuations and photobleaching in single-molecule experiments. Addressing these challenges calls for intensified research efforts focused on developing fluorophores with larger photon budgets,^80^ improved photobleaching resistance,^60^ and self-healing properties.^140^ Furthermore, advancements in fluorophore-coupling chemistries may facilitate orthogonal and turn-on labeling strategies that can be activated through caging-group-free light stimulation^180,181^ or ligand exchange.^236,327^

Future innovations that integrate techniques such as protein-induced fluorescence enhancement,^328^ photoinduced electron transfer,^329^ fluorescence correlation spectroscopy,^330^ fluorescence polarization,^331^ and fluorescence lifetime^332^ are anticipated to deliver complementary correlative information,^333^ enabling multidimensional insights into biomolecular dynamics, interactions, and conformational changes. The ongoing development of fluorophores and their integration with bioorthogonal chemistry will fulfill their promise as a foundational and transformative pathway for advancing single-molecule biophysics.

## Vocabulary Section

Photobleaching: A photochemical reaction between the excited electronic state of a fluorophore and molecular oxygen, resulting in an irreversible, single-step drop of its emission signal to the background level.
Photostability: The total number of photons that can be detected from a single fluorophore prior to photobleaching.
Photon budget: The total number of photons emitted and detected from a single fluorophore before photobleaching occurs.
Brightness: The number of photons emitted and collected per second upon excitation under defined conditions.
Photoswitching: The light-induced reversible transition of a fluorophore between fluorescent and nonfluorescent states.
Single-Molecule Localization Microscopy (SMLM): A super-resolution imaging technique that overcomes the diffraction limit by generating high-resolution images based on the precise localization of individual fluorescent molecules.
